# Atresia of the Genital Passages of Women

**Published:** 1880-09

**Authors:** Edward W. Jenks

**Affiliations:** Professor of Medical and Surgical Diseases of Women and Clinical Gynecology in Chicago Medical College


CHICAGO MEDICAL

JOURNAL & EXAMINER.

VOL. XLI.—SEPTEMBER, 1880.—No. 3.

### Original Communications.

ARTICLE I.

ATRESIA OF THE GENITAL PASSAGES OF WOMEN. A paper read before the Chicago Medical Society, July 19th, 1880. By EDWARD W. JENKS, M.D., LL.D., Professor of Medical and Surgical Diseases of Women and Clinical Gynecology in Chicago Medical College.

"*Les examples persuadent bien mieux que les simples raissonnements, et l'experience donne la perfection à tous les arts.*"—*Mauriceau.*

*Definition.*—The term atresia, of Greek derivation (ἀ privative and τρῆσις, perforation) and meaning in its literal sense an imperforate condition or entire absence of a canal or orifice, is by custom or conventionally used more liberally ; thus, atresia is the term sometimes made use of to designate partial obliteration of a canal, *e. g.*, atresia vaginæ, meaning literally an absence or entire obliteration of the vagina, but custom allows this term to be applied to a condition of the vagina where even the principal part of that passage is perforate, if only a little of it be imperforate. Atresia will therefore be made use of in this paper not in its literal but in its customary or conventional sense.

*History.*—In reviewing the history of atresia we find earliest mention made of it in the writings of Hippocrates and Aristotle. Hippocrates, in discussing the diseases of women, makes frequent mention of "obstructed uterus" and "closed neck." He uses the terms rather freely, it is true, but in one instance at least there is no doubt that he is dealing with a case of atresia. He says: "After accouchement the genital parts are sometimes closed by adhesions. I have also observed this when the orifice of the genital parts was ulcerated. Phrontis experienced what women suffer in whom the lochial discharge does not take place from this cause, furthermore she suffered pain in the parts, and on touching them observed that there was occlusion; she mentioned this, and upon treatment the lochia made its appearance; the woman was cured and remained fertile." *

Aristotle furnishes us with more precise details. He points out the origin of atresia, whether accidental or congenital, the possibility or impossibility of cure and the accidents which ensue with the arrival of the catamenia. "There are some women in whom from birth the os uteri is somehow closed; in others this is the result of disease; some of these cases are quite remediable, others not at all." Again: "In some women the os uteri is closed from birth up to the time when the menses are established, then with violent pain the os is torn open by the menstrual impulse, but with other women the obstruction must be dissected away by the physician; some women die after the obstruction has been forcibly removed or its attempted removal has failed."

Celsus has surpassed all his predecessors in describing means for overcoming atresia; he recommended first a crucial incision through the membrane and then its entire excision. For dressing he used a kind of tent, for which he substituted later in the treatment a leaden pipe (*fistulam plumbeam*).

Pliny cites the case of Cornelia, the mother of the Gracchi, as furnishing an example of atresia interfering with delivery.

Aëtius classifies atresias, dividing them into three varieties, according to the location of the obstacle: first at the labia, second in the vagina, third at the mouth of the uterus. He also desribes

* Littré's Hippocrates, Paris, 1855–61.

means for overcoming each of these varieties and enters into minute details of the subject.

Such then was the condition of affairs when science sought refuge with the Arabian physicians, and such it remained in their hands. That they did but little to advance the knowledge of this subject is largely due to the character of the Mohammedan laws, which forbade the examination of women by men, and hence the practice of obstetrics and gynecology fell almost entirely into the hands of the midwives. Avicenna, however, classified the varieties of atresia according as they interfered with menstruation or delivery; he also described a method of overcoming atresia by tearing an opening with the finger as well as the older methods of operating with a cutting instrument.

At the end of the fifteenth century Benivieni drew attention to to the first observation of imperforate hymen. In the sixteenth century Wier, Fabricius ab Aquapendente, Cabral and Felix Plater imitated Benivieni by inserting in their works facts which Jean Schenck collected some years later; although they form a compiled work, still the "*Observationes Medicæ Rariores*" is a book of great merit, and it is to be regretted that it was not more frequently consulted; by taking no notice of it many writers condemned themselves to mutual repetitions and barren paraphrases; still facts multiplied and happily observation continued to have its followers. Bauhin, Ruysch and Bartholin made up a contingent that surgeons, and especially *accoucheurs*, ought to extend their knowledge, citing with grateful recollection the names of Guillemeau, Dalechamps, Mauriceau, and Roonhuysen.

Still, notwithstanding the impulses given to the work by these men, progress in this branch of surgery was slow enough, bibliographers displayed no great zeal in research, and the most eminent surgeons were careless of recording success or failure in their operations. This condition only reached an end with the middle of the eighteenth century, and to Heister is due the honor of taking the initiative in the reform which followed. Instead of imitating his predecessors, he collected facts and wrote, so to say, at their diction the two chapters which he devotes to this affection. Following him a host of writers have touched upon the subject, but they have all been surpassed by Boyer, whose method and perspicuity are inimitable.

Since then the subject has been treated anew, and at greater or less length, but still with a deplorable slothfulness for research. In a subject where personal experience is necessarily limited, and where conclusions cannot have the force of laws unless based upon a considerable number of facts, we are often content with the precepts furnished by learning easy of acquisition, and we believe that our own task is finished when we have gathered together a score of cases, but these are not the means by which we carry conviction, or by which we definitely establish a course of operations both difficult and eminently dangerous.

Nevertheless, if our age has been disdainful of research it has certainly not made sport of such material as has come in its hands—it has been registered by the press, and been given a broad publicity, and it has known how to realize notable progress in an operative point of view; cases reputed hopeless have been attacked with success and bold surgery has reached its utmost limits.

Puech, to whom I am greatly indebted for much pertaining to the history of my subject, is of the opinion that Amussat and Debrou are the two names distinguished above all others among moderns in the treatment of atresia.

Coming down to our own day, and without particularizing individual achievements, we will merely add that the brilliant results which have been achieved in this branch of surgery in our own and other lands are not confined to a limited few; their names are legion, hence a complete modern history of the surgery of atresia would make too lengthy an article, and include a long list of names, many distinguished and some obscure.

*Varieties of Atresia.*—Of atresia of the generative passages of women we recognize the three following varieties in accordance with their localities: (1) Vulvar, (2) Vaginal and (3) Uterine. Any one of these varieties may be congenital or accidental, also complete or incomplete. The three kinds which have been named may be associated together in one individual, either congenitally or from traumatic causes.

*Vulvar Atresia.*—(*a.*) The labia majora may be adherent, but on account of their conformation and relative anatomy such adhesions do not prevent the exit of the menstrual blood, but sometimes do interfere with micturition and then calculi are formed, which of themselves require surgical interference.

(*b.*) The adherence of the labia minora like the same condition of the greater lips is usually the result of accident or disease, giving rise to the same difficulties of voiding urine. Unlike adhesions of the labia majora, adhesions of the labia minora may cause retention and accumulation of menstrual blood.

Atresia of both the greater or lesser lips may be consequent upon small pox, measles, or any constitutional or local disorder that can cause inflammation and denudation of their mucous surfaces. Such occurrences are without doubt more common in infancy and childhood.

(c.) Atresia of the hymen is one of the forms included under the heading of vulvar atresia, although not usually so designated, but it is generally spoken of as an imperforate condition. Imperforation of the hymen is of more frequent occurrence than either of the other forms of vulvar atresia. M. Puech*, who has written an excellent monograph on the subject considered in this paper, mentions one hundred and fifty-one cases, of which some were simple and some complicated with atresia in other parts of the genital passages. This author has observed seven cases, and from various sources has collected accounts of one hundred and forty-four others, making the above total of one hundred and fifty-one. He does not include in his list any cases ocurring prior to the age of puberty, and states that if such were to be added, one would be authorized in regarding imperforation of the hymen as an abnormality the most common. Emmet†, with his vast experience, mentions having met with only four cases of retention of the menses due to an imperfect hymen.

II. *Vaginal Atresia.*—This form may be congenital or accidental, as is the case with the other atresias, and also, like them, may be complete or incomplete. Courty ‡ and Puech § recog-

---

* De l'atresia des voies génitales de la femme par Le Dr. Albert Puech in 4° 165 p. Paris, 1864.

† Principles and Practice of Gynecology, by Thomas Addis Emmet. Philadelphia, 1869; page 211.

‡ Courty, Traité Pratique des maladies de l'uterus, des ovaries et des trompes. Paris, 1872.

§ Op. citat, page 11.

nize three varieties of congenital atresia, viz: simple, complicated and complex. They are called simple when the vagina alone is involved; complicated when there is atresia of both the vagina and the neck of the uterus; and complex when the vagina, being double, one of the canals is imperforate. The comparative frequency corresponds to the order in which they are here named, the last one mentioned being extremely rare. Its rarity can be readily inferred from the fact that Rokitansky observed only three cases among sixty-two of congenital vaginal atresia, all of the subjects being adults.

Courty mentions the infrequency of this form of atresia, and states that "none have been observed except by M. Leroy, M. Rokitansky and M. Décés."

There are probably some unrecorded cases, and some that Courty has not heard of. The writer has seen one case in the person of a Polish woman, who came to his clinic to be treated for uterine disease. She was single and twenty-four years of age. I am of the opinion that the adhesion which was in the left vagina might have been from vaginitis, as it was very easily made perforate, after which her double vagina was several times shown to the class, and the atresia examined by touch and by speculum, sometimes through one vagina and sometimes through the other. Some of the peculiarities of this case will be further spoken of when describing the cases which I have tabulated as coming under my own observation.

III. *Uterine Atresia.*—Atresia occurs less frequently in the uterine canal than in any other part of the generative passages. From all published accounts obtainable by Puech, the grand total sums up only fifty-six cases. There is not the least doubt that there have been many cases, recorded and unrecorded, which were unknown to this writer, especially in our own country, where surgical gynecology has had so many valuable contributions.

The same might be said of the statistics of Puech as regards other varieties of atresia already mentioned, and the first thought regarding them might be that they are of but little importance, but, although incomplete, they are none the less valuable as showing the relative frequency of the different varieties of atresia.

Nonat *, in his chapter on "Imperforations, or complete congenital atresia" of the uterus, states that "sometimes there is seen the opening of the mouth of the womb, completely masked by a sort of membranous diaphragm, being a dependence and prolongation of the mucous membrane which covers the lips of the uterine neck. When this membrane is obliterated, the cervical canal is found of the normal caliber."

J. Cloquet † calls attention to this fact, also, by stating that "Ruysch reports the case of a woman in labor three days without advance. The head could be felt in the vagina, covered with a membrane of great density, which closed the vagina. Ruysch incised the hymen, without any result. He then divided the second membrane, which was situated more deeply when the accouchment was rapidly and happily terminated."

The obliterations are, as a rule, limited to the interior portion of the neck. After diligent search I have been unable to find on account of a single instance where there existed an obliteration above the os internum, or even of the entire isthmus.

Atresias are first distinguished according to their location, and secondly all are included under the division of congenital, or accidental. By most writers the congenital are called imperforations, while the accidental cases are termed obliterations.

*Etiology and Pathology.*—Of the etiology of congenital atresia or imperforation it can only be said that there is arrest of development. The arrest in the complete atresias occurs in the early part of embryonic life. In vaginal atresia it often happens that arrest of development seems to be confined to the canal alone while the other genital organs develop as usual. This arrest of development may be complete or partial and in either instance the vagina fails to become a distensible canal. In some instances there is complete atresia, but the development occurs notwithstanding the existence of imperforation. Under such circumstances by rectal examination the vagina can be felt as a hard fibrous cord. The presence or absence of this cordlike vagina is of major importance as bearing upon prognosis and treatment.

* Nonat, Mal. de L'uterus. Paris, 1869; page 103.

† Dict. des Sciences Médicales. Bruxelles, 1829; art. Imperforation.

The etiology of congenital atresia is explained by Puech and others about as follows:

The female generative organs are developed in three zones. The internal zone has its origin in a blastema lying close to the Wolffian body. The ovaries are developed along its internal border, the Fallopian tubes along its external border, while the uterus is formed where the two borders meet, constituting the "genital cord."

The external zone is developed from a tubercle which makes its appearance at the caudal extremity. This, at first a simple elevation, is bisected by a groove, which deepens until it becomes a *cul-de-sac*, forming the cloaca. As for the tubercle, after dividing, it forms two slight elevations on each side of the groove, the upper two of these uniting above but remaining separate below form the labia majora and the clitoris, while the two inferior elevations develop into the labia majora.

The intermediate zone, from which the vagina is formed, is developed in the blastema, situated between the rectum and the bladder, immediately above the median perineal aponeurosis. This developes into a canal running from the vulvar opening to the neck of the uterus.

In cases of imperforation of the neck of the uterus the origin of the trouble is the same, development having stopped in the internal zone; exception being made in cases where the obstruction is due to a reflection of the mucous membrane of the vagina over the neck of the uterus.

Of accidental atresia or obliteration of the vagina there are pathological causes easily discerned. Injury or disease being the only causes, it simply remains in individual cases to determine the character of the injury or the relationship between obliteration and some co-existing disease. A vagina fully developed may be closed from the adhesion of its walls or its caliber may be diminished, constituting partial obliteration by the reparative process, which succeeds sloughing or true vaginal ulceration. I have myself seen three cases of almost complete vaginal atresia, complicated with vesico-vaginal fistula. There was a very marked similarity in the history of these three cases. In each one the fistula and atresia were in consequence of inflammation and sloughing following delayed labor and the unskillful delivery of a dead child. Two cases are reported by Courty* of accidental vaginal atresia associated with vesico-vaginal fistula.

Prolonged and difficult labors or the improper use of obstetrical instruments are among the most frequent causes of this form. Partial or complete obliteration may also follow easy non-protracted labors, and where instruments are not brought into requisition, yet in consequence of impaired vitality inflammation and loss of substance of the vagina ensues and subsequently atresia. Chemical agents locally applied to the uterus or vagina in the treatment of diseases of those parts, syphilis, vaginitis from any local or constitutional cause or any disease which produces ulceration or sloughing of the vagina are among the causes of this affection. Puech mentions three cases resulting from attacks of cholera. Courty† states that excessive and improperly performed coition, diphtheria, typhoid fever, caustic applications to the neck of the uterus, and particularly small-pox are some of the causes of atresia. Thomas‡ makes mention of a vaginal atresia produced by the passage of a sharp stick of wood, also of one caused by the retention for two hours of the trunk of a dead fœtus, the head having been previously delivered. This same author also alludes to an excellent article written upon this subject by Dr. Trask, based upon thirty-six cases, fifteen of which were due to prolonged labor. I have myself treated two cases of complicated vaginal atresia caused by the improper use of obstetrical instruments in childbirth.

There are some peculiar differences between congenital and accidental atresia and notably the following: If an atresia of the vagina is congenital, the canal is usually adherent throughout its entire length. On the contrary if it is accidental only a portion of the canal will, as rule, be obliterated; the upper extremity, the lower extremity or the middle portion of the vagina will be imperforate, while the remaining portions will be perforate.

*Symptoms and Prognosis.*—Vulvar atresia, with the exception of that form where the hymen alone is involved, is, as a rule,

* Op. citat., p. 398.
† Op citat.
‡ Diseases of Women. By T. Gaillard Thomas. Philadelphia, 1874, 4th edition. p. 162.

discovered in early childhood. On the other hand, attention is not usually called to the remaining forms until there are some pronounced symptoms indicative of their existence, such as inability to perform the marital act, or delayed menstruation, the patient being considerably beyond the age at which the catamenia is usually established. It has frequently happened that girls have for a long time, even for years, been under treatment for amenorrhœa until health had disappeared, when finally an atresia was found to have been the sole cause of the non external appearance of the menstrual flux. A girl may have the *molimen menstruale* and yet the flow of blood from the mucous surface of the uterus which occurs coincidently, instead of being discharged from the genital passage is retained by means of an imperforate hymen, and sometimes for a long period without the general health being affected. It is when the accumulation becomes so great that an additional amount threatens to rupture the Fallopian tubes or uterus, or be forced through the tubes into the peritoneal cavity, or there is evidence of blood poisoning that the health begins to be impaired. A physical examination under any of the circumstances just mentioned is demanded and should be insisted on. With any patient when there are indications of atresia in any part of the genital passages with impairment of health it is a duty on the part of the physician to make a physical examination for the purpose of ascertaining whether any local cause for the symptoms exists. Besides this there are often in such cases other questions hinging upon the physician's opinion aside from mere health. There are questions of happiness or misery to the individuals and families involved, if marriage is contracted, when the woman has any abnormalty about the sexual organs. This topic needs no elaboration. The duty of the physician is obvious, plainly so. One point I would insist upon, is that marriage or even an engagement should not be permitted if the woman has never menstruated, unless competent medical opinion sanctions it after thorough physical examination. On this particular point, a case of interest to myself at least, came under my observation. A young lady of an excellent family, while away from home, became engaged to a young man whom she met for the first time on the occasion of the visit. Soon after returning to her own home her mother wrote to the young lady's *fiancé* that there were reasons why the engagement should not be continued until medical opinion authorized it. The girl had never menstruated, although well developed in person, in fact the external organs of generation were perfect, even the mammary glands being of more than usual size. Several medical gentlemen of eminence were consulted, and all of them concurred in the opinion that marriage was allowable. She did marry and just nine months from the day of marriage, gave birth to a healthy boy, and has since had other children, but her menstrual history has been peculiar. She menstruated for the first time after she had weaned her first child. Menstruation has never lasted for an entire day, but usually only during four or six hours. Except when pregnant or nursing, it has been regular, unattended by pain, sometimes quite profuse in quantity, yet never of longer duration than before mentioned.

The number of patients with atresia of the genital passages of all varieties which have been treated by me is seventeen. Of this number eleven were congenital, and six were accidental. For the purpose in illustrating what has already been said, and what still remains to be considered within the limits of this paper, the cases referred to are here tabulated as follows:

TABLE OF CASES OF ATRESIA OF GENITAL PASSAGES OF WOMEN.

| *No.* | *I. Congenital Variety.* | *Age.* | *Remarks.* |
|---|---|---|---|
| 1 | Vulvar atresia of labia minora. | 4 | Operation and cure. |
| 2 | Vulvar atresia of labia minora. | 12 | Operation and cure. |
| 3 | Vulvar atresia of labia majora. | 14 | Operation and cure. |
| 4 | Imperforate hymen. | 10 | Operation and cure. |
| 5 | Imperforate hymen, with vaginal walls adherent for an inch and a half in the inferior portion of the canal, constituting true vaginal atresia. | 20 | Signs of blood poisoning from retention, the vagina being distended with menstrual blood so that fluctuation was plainly discernible. Operation successfully made. |
| 6 | Complete vaginal atresia. | 22 | Operation begun but not completed, for reasons given in the text. No menstrual accumulation. |
| 7 | Double vagina. Complex vaginal atresia. | 28 | Atresia of one vagina, probably from vaginitis. Dilation easily accomplished. |
| 8 | Absence of uterus. | 34 | Patient married; was subject to epileptic convulsions occurring every four weeks. |
| 9 | Complete uterine atresia. | 20 | Patient single. Case reported in one of my clinical lectures in *Obstetric Gazette*, January, 1880. |

TABLE OF CASES OF ATRESIA, ETC.—CONTINUED.

| No. | I. Congenital Variety. | Age. | Remarks. |
|---|---|---|---|
| 10 | Partial vaginal atresia, interfering with labor. | 25 | Labor could not be completed until the parts were incised. |
| 11 | Atresia of the upper portion of the vagina. | 30 | Patient married. A rudimentary uterus could be felt very indistinctly per vaginam, but plainly per rectum. No menstrual accumulation. |
| *No.* | *II. Accidental Variety.* | *Age.* | *Remarks.* |
| 12 | Partial vaginal atresia in upper portion of canal, caused by application of caustics to the uterus. | 31 | Patient married. Uterus could barely be touched with the finger. Operation and cure. |
| 13 | Partial vaginal atresia, with vesico-vaginal fistula. | 29 | First operated upon for atresia, the vagina barely admitting the little finger—a second operation was required for the fistula. |
| 14 | Partial vaginal atresia, with vesico-vaginal fistula. | 19 | First operated for atresia, which occupied the middle portion of vagina, not permitting a view of the fistula or cervix uteri. Afterwards operated for the fistula, which was bounded on one side by the cervix uteri—urethra obliterated. |
| 15 | Partial vaginal atresia, with vesico-vaginal fistula. | 27 | This case was similar to the above one except as regards the obliterated urethra. |
| 16 | Vaginal and uterine atresia or complicated atresia. | 30 | Caused by protracted efforts on the part of several physicians in delivering the patient of a dead child at full term. Following delivery there was severe pelvic inflammation, pelvic abscesses, and a discharge of pus for months. The upper portion of the vagina and the cervix uteri were occluded. Menstrual accumulations were found, and alarming symptoms occurred at menstrual date from threatened rupture. Successful operation. (*Detroit Review of Medicine*, June, 1876.) |
| 17 | Vaginal atresia, with large uterine fibroid. | 38 | Reported in *Chicago Medical Gazette*, in March, 1880. |

### COMMENTS ON THE FOREGOING CASES.

It will be observed that in the above list numbers 1, 2, 3 and 4 were quite young subjects, and consequently no serious trouble had resulted from the abnormality. But little comment is necessary in connection with them. The discovery of the malformation, whether accidental or resulting from the watchful care of mothers, led to speedy cure before the occurrence of menstrual accumulations. Two of the number, however, had attained the age at which the menstrual function is usually established, while number three had, previous to the surgical operation, experienced the *molimen menstruale* very markedly and menstruated soon after.

I shall briefly allude to the remaining cases at greater length than in the remarks of the tables, but will not in every instance follow the order in which they are tabulated but in such sequence as seems proper from their mutual relations.

Case number 5 was a domestic, who had been dismissed from employment and denied admission to the homes of her friends because of the belief that she was pregnant. One lady, however, by whom she had been employed requested her own physician to see her and determine the cause of her enlarged abdomen. This physician, who unfortunately was not a regular one, but one who was accredited with great shrewdness, declared the girl in the latter months of pregnancy, and so the poor girl was again sent out in disgrace. A benevolent gentleman, and one of the most distinguished public men of Michigan, learning in some way of this girl, and, believing the story of her innocence to be a true one, sent his housekeeper to provide a place for her, and desired me to ascertain the cause of her peculiar appearance. I found, as I then believed, an imperforate hymen only, and by rectal examination an accumulation of menstrual blood. When I came to operate in this case, however, I found more than an imperforate hymen—there was atresia of the lower portion of the vagina to the extent of an inch or more. After incising there was a discharge of several ounces of menstrual fluid of the usual tarry consistency, whereupon the vagina and uterus were syringed out with warm water. The operation itself requires no special comment in this place; suffice it to say that it combined the tearing and cutting processes, followed by the use of vaginal plugs until the parts were healed.

The patient numbered 6 in the list was a young lady twenty-two years of age, who had been treated by regulars and irregulars, of all kinds and creeds, during a period of several years to "bring on her courses." Among the multitude of her medical attendants not one had suggested the propriety of making a physical examination until she became the patient of Dr. G., a painstaking and skillful physician of the town where she resided. I was called to see the patient with him, and we discovered the cause of the amenorrhoea to be a complete vaginal atresia, but there was no accumulation of menstrual blood. The external organs of generation exterior to the vagina were well developed as were also the mammary glands. The mons veneris was covered with hair, which is not usually the case unless the ovaries are developed. By examining per rectum a uterus could be felt, although quite small. By means of a sound in the bladder and a finger in the rectum the vagina could be plainly felt as a large round cord. An operation to construct a vagina, or rather to dilate the rudimentary one already existing, was proposed and begun by carefully opening the canal for about an inch and a half and using tents to keep it open. This was prior to the adoption of the mode of exploration by dilating the urethra and examining the interior of the female bladder with a finger. I deemed it my duty to say to the mother of the girl that marriage, under the circumstances should not be thought of, but that if the operation ultimately succeeded then would be the time to decide the matrimonial question. The girl, it seems, was engaged, but was willing to wait until the propriety of marriage could be decided by the success or failure of the surgical procedures. The lover, however, learning the true state of affairs, suddenly emigrated to a milder climate, and the girl herself was so disgusted with his course that she pulled out the tents from the partially constructed vagina and vowed to lead a life of celibacy. Consequently what might have been accomplished in her case will never be known. In my own mind there is no doubt that complete success would have attended the operation in due time, that is to say, a vagina would have been opened, not constructed, in evidence of which was the fact that the cordlike vagina could be so distinctly felt.

Patient No. 7, with a double vagina, has been sufficiently spoken of under the head of vaginal atresia. This case represents that rare form of artesia designated by Puech, Courty and others as complex, viz: a double vagina, with one canal imperforate.

Case No. 8 was referred to me by Dr. Dayton, of Lima, Indiana, with whom I saw her, and from whom I learned the peculiarities of her medical history. She was thirty-four years of age, a brunette of spare form, and had been married for several years, the exact number I did not learn. Following her marriage she had become subject to epileptic convulsions; at least they were so designated, but they may have been merely epileptiform, or hystero-epileptic. These convulsions occurred once a month, and coincident with a marked *molimen menstruale*, and occurred at no other time. Her external organs of generation were well developed, giving sufficient evidence of fully developed ovaries. She acknowledged possessing a keen sexual appetite, and her husband stated that it was at times rather aggressive. The vagina was simply a pouch of normal length, but without the vestige of a uterus to be felt within or beyond it. But by rectal exploration a rudimentary uterus of minute size could be plainly felt, as could also the converging round and broad ligaments. As it was manifest that the epileptic or epileptiform convulsions occurring as they did coincident with the *molimen menstruale*, were of ovarian origin, I proposed Battey's operation as affording the only possible means of relief, but the patient would not consent to it, and, soon after, passed beyond my observation.

Patient No. 9: An account of this case was published in the *Obstetric Gazette* for January, 1880, as a clinical lecture, by Dr. Helms. The patient alluded to, an inmate of Mercy Hospital of this city, was a girl of twenty, with perfectly developed external organs of generation, the mammæ, indeed, being larger than common. The patient longed to be relieved of the distressing symptoms which marked her case, and which occurred once a month, such as congestive headache, severe pelvic and abdominal pains, etc. These symptoms had of late been progressive, and had attained such severity as to be alarming. There was no accumulation of menstrual blood; the vagina was of normal appearance, size and length, but the cervix uteri appeared by speculum examination as a mere nodule, without the least indication of a uterine canal. By rectal examination, the uterus was found to be about three-fourths of an inch in length, thus indicating arrest of development with atresia. This patient, as before remarked, was the subject of a clinical lecture, and the condition of things as described was shown to my class. As no uterine canal could be found by ordinary means, I clipped off with scissors a small fragment of the rudimentary neck, but no further signs of a canal were discovered; still this procedure proved of great service, as the loss of blood near the time of approaching *molimen* materially lessened her sufferings. The only chance of a radical cure seemed too formidable to advise until milder means had been exhausted. The radical cure was removal of the ovaries; but, from the fact that the abstraction of blood immediately preceding the *molimen menstruale* had so greatly alleviated her sufferings, I advised the periodical abstraction of blood, by means of leeches applied to the perineum. As long as I continued to hear from the patient, this plan proved successful.

Case No. 11 was in the person of a primapara whom I attended in labor. She was a woman of unusual size, with a correspondingly large pelvis. Labor progressed favorably until the child's head was in the pelvic excavation, when it stopped. Making a careful examination, I now discovered that there was partial atresia of the lower portion of the vagina. Here the obstructing membranes being put upon the stretch by the process of labor gave the appearance of a broad band, and, while in this tense condition, I made an incision through them fully three inches in length. The incision once made, laber was completed in a brief space of time. The atresia was simply a semilunar-shaped hymen, which, not having been previously ruptured, remained an obstacle to the free egress of a full-sized fœtus.

The case of patient No. 11 presented some similarities to that of No. 8. The remarks in the tabulated list in connection with this case comprise about all that is requisite for this paper. This woman consulted me for the purpose of ascertaining whether or no she could be successfully treated for sterility. She was thirty years of age, of spare frame. The external organs of generation were not perfectly developed; the labia presented a shriveled appearance; the *mons veneris* was scantily supplied with hairs; the mammæ were small, with but little gland structures; the vagina was sufficiently capacious, but not of usual length, ending in a blind pouch, which gave no appearance of a uterus, which organ was only discoverable by firm pressure in the hypogastrium, or by rectal examination, in either of which cases a rudimentary uterus could be readily felt. The case was simply one of partial vaginal atresia of this congenital variety, with non-development of the uterus. I could not advise or institute any surgical procedures or treatment, as there were no urgent symptoms making such demands.

The remaining cases which I have tabulated are all non-congenital, the results of either injury or disease.

Patient No. 12 consulted me on account of uterine disease, for which she had already been receiving treatment during the previous two years. This was at a time when all real or fancied uterine diseases were subjected to the application of caustics. Such had likewise been the local treatment in the case of the patient now under consideration. She said that the stick of caustic had often been left in the mouth of the womb ; that the treatment had been very painful, and particularly the last time the caustic had been applied, when a large stick of it had been used. For some days she was unable to walk, and finally a discharge of pus and blood set in. Three or four months had now elapsed since the last application, but, although the painful symptoms had subsided and the discharge of pus ceased, she was conscious of something abnormal, as coition could not be perfectly performed, and all attempts caused severe pain and oftentimes discharge of blood. An examination revealed the vagina almost obliterated in the upper portion, so that a finger could with great difficulty be made to force its way to the cervix uteri, while the cervix itself was bound to the left side of the vagina by firm adhesions. The cause of this partial atresia was, without doubt, the free use of caustic, which was dissolved in the vagina instead of the uterus. A cure was effected by means of cutting with scissors a portion of the adhesive bands, and by constant use of vaginal plugs.

Cases 13, 14 and 15 will receive but little comment, as a brief allusion will be sufficient. A full and succinct history of each one would be of more interest as relating to vesico-vaginal fistula than to the subject under consideration. Every one familiar with vesico-vaginal fistula knows that one of the most frequent complications met with is that produced by the presence of cicatricial tissue, which is often in the form of bands of greater or less dimensions. It may be considered an unusual case, as far as my own experience goes, that has no cicatricial bands in the vagina. The patients here referred to, however, had more than cicatricial bands, in consequence of the causes producing fistula. There resulted, in each case, almost complete obliteration of the vagina, fully entitling each one to be designated as incomplete vaginal atresia. In No. 13 the little finger could with difficulty be crowded into the vagina, requiring two operations preliminary to the one for the fistula, and also the use of dilators. Not until after the first operations had been performed could either the neck of the uterus or the opening into the bladder be felt or seen. Nos. 14 and 15 were similar to the case just mentioned, there being vaginal obliteration to such an extent as to necessitate an operation before the fistula could be seen or felt.

Patient No. 16 was a case of unusual interest to the writer, at least. It was one of those cases of which it is extremely difficult to describe all the particulars so that one not seeing and examining the patient can scarcely get a clearly correct opinion of the complications and difficulties of the operations made for her relief. I am not alone in this opinion, for several gentlemen present at the time I operated expressed themselves similarly. Among this number was Dr. Johnson, of Detroit, a former pupil and assistant of Simon, of Heidelberg, and Dr. H. O. Perley, Asst. Surgeon U. S. A., at that time a resident of Detroit. An account of the case was published in the Detroit *Review of Medicine* for June, 1876, from which I will give a brief abstract: *

"Mrs. C. of Ohio, consulted me on January 12, 1876, with reference to an operation for atresia, which she stated was caused by the delivery of a dead child May 10, 1875. She was confined to her bed for six weeks after delivery on account of inflammation of the womb and vagina; subsequently abscesses formed within the pelvis, and discharged externally from time to time up to the date of her visit to me. Three months after delivery there were symptoms indicating the appearance of the menses, with some constitutional disturbance. A month later there was again the *molimen menstruale*, with increased pain and more general disturbance; the pain was progressively severe with each return of

---

* "Atresia vaginæ et uteri; produced by instrumental delivery of a dead child; operation and cure." By Edward W. Jenks, M.D., Professor of Medical and Surgical Diseases of Women, Detroit Medical College, Detroit. *Review of Medicine*, June, 1876.

the menstrual date, and had become so alarming that, at the time of her visit to me, both she and her husband earnestly desired that some step be taken for her relief before the return of another attempt at menstruation. * * * * * Physical examination of the generative organs revealed a laceration of the perineum to the margin of the sphincter ani externum; the vagina was closed superiorily, nor could any portion of the uterus be felt in this canal, neither could I, by any means of speculum and probe, find any trace of the upper part of the vagina in the abundant cicatricial material which obliterated it. It seemed at first as if the vagina was about the normal length, and that it was only a short distance to the neck of the uterus, and yet it could not be felt. This state was further explained by a rectal examination. By the rectum the body of the uterus was felt very high and retroverted, and apparently held in that position by firm adhesion, as if the inflammation had extended to the pelvic peritoneum simultaneously with the vaginal and uterine inflammations. Besides this the vagina was an unusually long one, and its apparent length at this time is explained by still another reason, namely, the great size of the patient, who weighed two hundred and eighty pounds. The abdominal walls, burdened with fat, rendered conjoined manipulation of little service in diagnosis or in the subsequent operation. The same condition of the labia majora made the vagina seem long, and rendered ocular examination more difficult than usual." * * * The patient being placed in the exaggerated lithotomy position, the pelvic organs were fixed by the pressure of an assistant's hand on the hypogastrium. I then, with sharp pointed scissors, carefully cut a portion of the cicatrix in the presumed direction of the uterus, occasionally ascertaining the safety of my incisions by introducing a finger into the rectum, or the sound into the bladder. I soon found my finger and sound in close proximity, showing the necessity of great caution lest I should cut either into the peritoneum or into the bladder. After cutting through upwards of two inches of cicatrical tissue I reached the uterus, but as there was no indication as to the locality of the os uteri I separated a portion of the neck from its morbid vaginal adhesions, but the os was not apparent, nor was there anything in the appearance of the enucleated neck to indicate the route into the cavity of the uterus, so I then cut with a pair of very delicate and sharp pointed scissors in the direction of the uterine canal for about an inch, when there gushed out between two and three ounces of menstrual blood. The uterine canal being now discernable, I enlarged the opening still more and cut away the cicatrix from the newly made os. A tent of lint was put in the uterine canal and the newly opened portion of the vagina packed with the same material, when the patient was put in bed. But little blood was lost during the operation aside from the menstrual blood, and when the patient was restored to consciousness she was kept free from pain by moderate doses of morphine per rectum. The next day the lint was removed and a fresh quantity put in with the finger, for the purpose of preventing union by first intention. The patient complained but little of pain, and after the removal of the first tampon there was but little oozing of blood. The fourth day after the operation word was brought me that Mrs. C. had a "chill," and complained of quite severe pain in the abdomen. It was several hours before I saw her, but when I did I was gratified to learn that the rigor and pain were simply the ushering in of the menstrual flow, and that she was now free from pain. The catamenial discharge continued five days, as was her former custom. This I considered a happy occurrence, so soon after the operation, as the danger of closure of the wounds, especially within the uterus, was by this opportune discharge greatly diminished. The patient rapidly recovered her usual health, and three weeks later returned to her home." * * * "In conclusion, there are certain points of interest in connection with this case to which attention is directed: *First.*—The dangers as marked by the symptoms of rupture of the Fallopian tubes or regurgitation of menstrual blood into the peritoneal cavity, if the operation had been much longer postponed. *Second.*—The complications from traumatic causes, viz: a retroverted uterus, bound down by peritoneal adhesions, obliterating a portion of the uterine canal, and the locality of the cervix uteri obscured by a great quantity of vaginal cicatricial tissue, to which may be added the difficulties attending any manipulation on account of the size and obesity of the patient. *Third.*—The advantage of operating just preceding the regular catamenial date, as was manifested in this case, by the menstrual blood keeping the newly opened canals pervious in a better manner than could be done by tents, or any other device."

Case number 17 was reported in the *Chicago Medical Gazette* in March, 1880, under the following heading: "Supposed Inversion of the Uterus—Fibroid Development of Posterior Half of Uterus and Vaginal Atresia." A full relation of the peculiarities of the case would contain much that is not pertinent to the subject of this paper. It will suffice to say that there was almost complete vaginal atresia, the perforate portion of the canal barely admitting a small sized probe. The atresia was overcome by lacerating or tearing with the finger, so that a diagnosis of the exact condition was easily determined. The spherical character of the tumor together with the atresia was what led to the belief that it was a case of inverted uterus. The atresia doubtless had its origin in vaginitis, which I judged from the history of the case as related to me, was caused by the application of caustics, employed for some reason past discovery in the treatment of the patient.

*Treatment.*—The treatment to which I have subjected my own patients has been set forth in the reports of individual cases, but the subject is of sufficient importance to be more fully discussed.

At the Baltimore meeting of the American Gynecological Society an excellent paper was read by Prof. Isaac E. Taylor, of New York, entitled, "Atresia of the Vagina, Congenital or Accidental, in the Pregnant or Non-pregnant Female." I was not present at the meeting, and, owing to delay in publishing the society's transactions, did not have the privilege of reading Dr. Taylor's paper until the principal portion of this one was written. Dr. Taylor's paper I consider a valuable addition to the literature of the subject, as his own experience has been large and varied. He has also drawn from the experience of others. The subject is one which has not been written upon very extensively, the literature consisting chiefly of the reports of cases. Dr. Taylor's paper possesses an interest in one particular, to which I have merely made brief allusion; that is the association of pregnancy with complete vaginal atresia. It does not discuss the whole subject of atresia, for he announces in the first clause that he desires to confine himself "to cases of complete congenital atresia and to accidental absence or atresia of the utero-vaginal canal in the pregnant and non-pregnant woman." This writer further states that, "Cases of congenital absence of the vagina are rare; cases of the same nature existing during pregnancy and involving two lives are still more so."

He relates a case of complete congenital atresia of the vagina, where the woman became pregnant and was safely delivered, at full term, of a living child. He also refers to a case of Dr. Simmons, extracted from the St. Louis *Medical Examiner* for February, 1847, of a similar character, and yet, in each one, the physicians were unable to find the minutest opening between the lower or more external portion of the vagina and the uterus. By Dr. Simmons this question is asked: "In what manner did conception take place?" Dr. Taylor, referring to his own case, and in the same connection speaking of Dr. Simmons', gives a "solution of the mystery." As the discussion of this question is not strictly pertinent to the present paper, it will not be further considered.

The treatment of all forms of atresia cannot be otherwise than surgical, and, with the exception of certain forms of partial atresia, all may be considered of great importance, requiring care, caution and skill. Even the operation for an imperforate hymen, so simple in itself, has many times proved fatal. Bernutz * lost four patients; Thomas † mentions two fatal cases, one of which, indeed, was his own. Sir James Y. Simpson collected the history of several cases where the operation proved fatal; while, scattered through medical literature in general, may be found the reports of many of these operations, which resulted in the death of the patient. Simpson mentions a case of death, in which there was occlusion of the vagina from adhesion, the septum being quite thin, but, as the menstrual blood was retained, a small incision was made, through which there poured out, during a number of days, large quantities of the usual dark grumous fluid. Surgical fever set in on the third day after the incision was made,

* Clin. Méd. sur les Mals des Femmes. Tome 1, p. 303.

† Diseases of Women. By T. Gaillard Thomas, Prof., etc. Phila., 1879.

and in a few days more the patient died. An autopsy revealed a distended uterus with inflamed walls, and inflammation extending thence to the peritoneum. The mistake made by the operator in this case was the small incision and neglect to wash out the cavity, as undoubtedly the entrance of air had caused decomposition, resulting in death from septicemia. This is a point which will be discussed later.

"It is conceded," says Dr. Taylor, "that, no matter what the exact nature of the obstruction may be, the accidents which cause death after operation are almost identical." While I would accept this as in the main true, it still seems to me that the statement requires modification.

There are three causes producing death, which are not identical in their nature; they are septicemia, inflammation and rupture; consequently, it seems to me, that it could with greater propriety be said that the deaths which occur in consequence of operations, no matter what be the nature of the obstruction, are generally due to septicemia, inflammation or rupture; in some instances to a combination of these causes, and occasionally to the shock of rupture alone, without the supervention of either septicemia or inflammation. Unquestionably septicemia ranks first as the most fatal, particularly after operations for vulvar or vaginal atresia. This being the case, the prophylaxis of blood-poisoning is of paramount importance, and should not be lost sight of in any surgical procedure, or in the after-treatment.

Owing to the dangers attending surgical operations of this variety, even eminent surgeons have objected to them, and advise non-interference; but such advice is not likely to be followed at the present time, when so much is being accomplished by antiseptic treatment. The propriety of surgical interference, or the necessity for it, can only be determined in each individual case. Where life or health is threatened, surgical procedure seems to be demanded.

There is a peculiar feature in the history of complete or incomplete atresias of the generative passages, which is interesting, and has a bearing on treatment in cases where there is an accumulation of menstrual blood. This feature is alluded to by Barnes.*

* Diseases of Women. By Robert Barnes, M.D., London, Lecturer on Obstetrics, etc. Phila. 1878; page 206.

When there is stenosis or atresia of long continuance, the genital canal "undergoes retrograde dilatation above the seat of the stricture. This is the almost inevitable consequence of the futile attempts of the muscular coat to expel the retained contents." This effect is seen in the most marked form in cases of imperforate hymen; the vagina, being the most distensible part of the canal, dilates first, forming a large pouch; then the cervix uteri is distended; then the cavity of the body of the uterus, and lastly the Fallopian tubes. This dilatation, conservative in its effects by accommodating the contents which cannot be evacuated, has its limits. When these are reached, the danger of rupture or perforation at the weakest point is great. Before such an occurrence takes place, the imprisoned fluid, by reason of the constant pressure to which it is being subjected, may be forced to ooze through the walls of the uterus in drops, causing septicemia or peritonitis. As analagous to this condition, the opinion is expressed by Barnes that in a similar manner some cases of pelvic peritonitis are produced. More commonly, in consequence of retained menstrual blood, equally grave results of a different kind may follow. The uterus, exerting great pressure upon the fluid, may force it into and through the Fallopian tubes, whence it escapes at their fimbriated extremities, or, it may be, forced through some weakened part, producing a rupture. Either of these occurrences are rendered easier on account of the continued dilatation of the tubes. What has just been stated has an important bearing upon operations for imperforate hymen. Again, the sudden evacuation of a large quantity of menstrual blood by a free incision may cause such sudden collapse of the uterine walls as to excite contraction of that organ, and, in consequence, some of the blood, by a regurgitating process, is forced through the entire length of the tubes into the peritoneal cavity, or else through the substance of the tubes themselves, rupture taking place at the weakest spot of the distended walls. It is thought by some that the more sudden the evacuation the more liable such occurrence. It is believed by many that the sudden admission of atmospheric air into the vagina is the cause of the uterine contraction, rather than the collapse of that viscus. A case is related by Béclard, in which the uterus burst, discharging into the bladder. Other writers have mentioned instances of rupture of the hymen. Puech refers to a case of spontaneous cure of an imperforate hymen, by reason of ulceration of the obstructing membrane. Another condition demanding surgical interference in cases of atresia is where the menstrual fluid, being retained there, is liability of blood-poisoning from absorption into the system of the changed blood. The patient in my own list, numbered 5, showed this condition in a pronounced manner.

It might be interesting to some of my auditors to hear the reports of peculiar cases and the opinions of distinguished authorities, on the treatment of the class of abnormalities under consideration, but it seems needless to me in this assembly of practicing physicians to relate what many are familiar with and what all can have access to in most of the late text books on diseases of women.

Without further discussion of these points, or the demands for surgical interference, the modes of operating and the treatment requisite to render operations successful will be considered.

The evacuation of retained menstrual blood can be accomplished with greater safety by means of an aspirator than by any other method. In cases of imperforate hymen the membrane should not be incised until it is first ascertained by rectal examination whether menstrual blood is present, and if found in considerable quantity, the fluid should be aspirated, and as soon as it is believed that all is thus evacuated, then a free opening should be made into the vagina, first cutting and then tearing; the vagina, and if possible, the uterus should be washed out with warm water until it runs out without discoloration. By so doing there is but little danger of regurgitation of the fluid and the risks of septicæmia are lessened. If the quantity is considerable, then the fluid should not all be aspirated at one time but at different times, for, if the contents are nearly all drawn away at once, the ensuing collapse excites the uterus to contractions which may cause the regurgitation or rupture previusly referred to. Small openings should never be made for the purpose of gradual drainage, as they afford ingress to air, causing subsequent decomposition.

Bermutz, who lost four patients from free incisions through the hymen, selected as the time for operating, the second week after menstruation, as the time when the organs are less liable to be disturbed.

In cases of children, some authorities have advised no interference until the age of puberty, but I am of the opinion that an imperforate hymen or vagina, the only form of atresia likely to be discovered in childhood, should be operated on and kept open by tents. In children incisions should be avoided if possible and the canal opened by laceration or tearing.

The means of opening the genital canal, whether the obstruction be in the vagina or in the uterus, are tearing or laceration, the scalpel, the scissors and the trocar. The aspirator can scarcely be enumerated as a means of opening an imperforate or occluded vagina or uterus, as its use is rather for evacuating fluid therein contained.

In some instances a combination of tearing and cutting serves the best purpose. As a rule I would advocate giving preference to the tearing process in congenital atresias, but it will be found impracticable in the accidental varieties. Emmet prefers scissors, as is well known to all familiar with his practice. My own choice for the vagina is scissors and for the uterus either very delicate scissors or a tenotomy knife. The tearing open of imperforate canals with the finger is usually the better as well as easier plan, but where union has occurred in consequence of inflammation or injury, tearing can be done only to a limited extent, for the reason that dense structures like cicatricial tissue will not readily tear and if the requisite force is applied one is liable to lacerate around rather than through it; the character of the obstruction should always determine the mode of overcoming it. The trocar I would avoid as much as possible, as about the only case where I deem it admissible is in uterine atresia. To those who practice one of the operations of Amussat the trocar is indispensible, but that operation, which consists of plunging the trocar through the rectum into the vagina for the purpose of evacuating the menstrual blood is, as a rule, unwarrantable. This may seem strong language, since for years this has been successfully practiced, but I firmly believe that the case must possess most unusual abnormality if menstrual blood cannot be abstracted along the route of the vagina, its natural and only proper outlet. The trocar has proved serviceable in atresia of the neck of the uterus, serving in some instances indeed a much better purpose for opening the canal than either the knife or the scissors. Syme* objects to the trocar and is in favor of the knife, which he uses with great freedom.

It is important, after the vagina or uterus is made perforate by operation, that some measures be taken to keep it so by means of tents, plugs or dilators. One case came under my observation where a physician had plunged a trocar through an imperforate hymen, a large quantity of restrained menstrual blood escaped, but the incision closed up and a subsequent operation became necessary.

It has been a matter of considerable discussion by many surgeons in cases of complete vaginal atresia how much should be done in a single operation. Amussat† in 1832, in operating in a case of congenital atresia, cut through the skin, and then from fear of entering either the rectum or bladder with his knife tore an opening for some distance with his finger and packed the orifice with a sponge; after three days he repeated the process, and subsequently again repeated it; on the tenth day he reached the tumor, which he emptied with a trocar. The patient suffered from severe inflammation of the Fallopian tubes, but after four operations with the trocar the canal remained sufficiently open. Emmet‡ advocates an opposite course from that pursued by Amussat, and cites cases where he accomplished at a single operation all that was accomplished by Amussat in ten days. In my own case, number 16, I unwisely followed the plan of Amussat for the same reason, but I have never ceased to regret that I did not attempt to reach the uterus at the first operation, as the peculiar circumstances in which the patient was placed was the means of making it also the last and only one. I am convinced that the vagina should be made perforate with as few operations as possible—and whenever it is practicable it should be accomplished in one operation, exception always being made in such cases as have been alluded to, where there is a large accumulation of menstrual

* Taylor, Op. citat.

† Gaz. Med. de Paris, 1835.

‡ Principles and Practice of Gynecology, by Thomas Addis Emmet, 2d ed. 1880.

fluid, and there is reason for thinking that considerable dilatation of the uterus or tubes exists. In a very recently published discussion on the method of removal of retained menstrual fluid, by members of the Obstetrical Society of N. Y.* a variety of opinions were expressed, some advocating rapid removal and others a slow process. Emmet expressed himself strongly in favor of a rapid evacuation, and as to the most unfavorable cases he believes them to be those of congenital absence of the vagina with a distended uterus. Dr. Lusk said that the danger was greatest from inflammation, if air entered a flabby vagina and uterus; while firm contraction removed danger. Dr. Mundé said the chief source of danger was from sepsis and regurgitation through the Fallopian tubes. Dr. Skene believed that the chief source of danger is inflammation; that death more frequently is caused by it than by septicemia; he would give patients to understand that the operation is a dangerous one, and in his opinion inflammation is the danger most to be feared.

Another undecided question is, whether it is better to operate before or after menstruation or the *molimen.* The history of my own case, number 16, leads me to give most pronounced favor to operations before the period. Where there is no accumulation, and the operation is chiefly for the purpose of relieving a deformity, then the time of greatest calm of the generative organs should be selected, which is ten or twelve days after the menstrual date. I do not believe it is wise to adopt a strict rule of practice however in regard to this matter. The pathological condition, as well as the exigency of the case, must be considered, thus in truth making each case a rule for itself. The same is perhaps true as regards operations for atresia in pregnancy, yet my own opinion, based solely on the experience of others, is that it is better to wait until labor has begun rather than to make any operation prior to the establishment of the labor pains.

The question has been asked, "When should uterine injections be resorted to?" It is claimed that their immediate use causes inflammation, but it is quite probable that such a condition has resulted rather from the manner of their use; even the forcible

---

*Amer. Journal of Obstetrics, July, 1880, pg. 609.

injection has been used, while some have employed astringent and medicated injections. The use of injections is of great importance for the prevention of septicemia. The manner in which they are used is of equal importance if it is the uterus that is to be washed out. It is not positively necessary that haste should be exercised in employing them, as no great amount of decomposition and certainly no blood-poisoning can occur in from two to six hours; but, as a rule quite safe to follow, the passage should be washed as soon as practicable after the atresia is overcome. I have in a former paper* expressed my own views and the opinions of recognized authorities on the propriety, the dangers and the manner of using intra-uterine injections. The same general rules are to be followed in washing out the uterus after operating for atresia as in any other condition or disorder. That no harm may follow their use, certain requisites are important, as follows: (*a.*) A free outlet should be insured for the injected fluid. (*b.*) Air must not be admitted with the injected fluid. (*c.*) Cold astringent or irritating injections should not be made use of under any circumstances within the uterus, as they are apt to excite contraction, in consequence of which the fluid is liable to be forced through the tubes, or cause rupture at the weakest point. (*d.*) Forcible injections are to be avoided, as the injected fluid may be forced through the tubes, or cause rupture at some point already weakened by dilatation.

In looking over all attainable literature on the subject of this paper, I have been impressed with the meagerness of it, and with the apparently unsettled views of the majority of writers in regard to treatment. There are very few exhaustive essays on the subject, the literature consisting chiefly of reports of cases, with fragments here and there in text-books and the medical periodicals, the most elaborate essay being the one of Puech before referred to. It is possible that one cause of the divided opinions regarding treatment may be due to the fact that, whatever the treatment adopted, cases prove fatal, and with the necessarily limited number coming within the observation of any one individual, each thinks of his serious or fatal cases that, if they had

* The Treatment of Puerperal Septicemia, etc. By Edward W. Jenks, M.D. Trans. Am. Gyn. Society, 1879.

been subjected to a different course of treatment than the one pursued, they might have been less serious, or even recovered.

In concluding this paper, I will, on the subject of treatment, only attempt to give a summary. From the literature of the subject and the reports of cases, to which may be added my own observation and experience, I am led to the adoption of the following conclusions:

I. As fatal results have followed operations for the simplest varieties of atresia, the surgeon should apprize the patient, or her friends, of every possible danger prior to operating.

II. In case of menstrual retention from vulvar, vaginal or uterine atresia the fluid should not be evacuated via the rectum with a trocar, but in the route pursued by the vagina.

III. When there is reason for believing that there is a large quantity of menstrual fluid distending the uterus or Fallopian tubes, the safest mode of evacuating it is by means of an aspirator prior to any surgical procedure for the cure of an atresia.

IV. The evacuation of menstrual fluid should never be through a small orifice (with the exception of aspiration), but through a free opening, after which the vagina and uterus should be thoroughly washed out with warm water, as the best means of preventing or curing septicemia or inflammation.

V. Septicemia, inflammation and rupture of the Fallopian tubes are the chief disasters attending atresia, or following operations for its cure.

VI. Congenital atresia of the vagina can be best relieved by tearing with the finger, as the rudimentary canal already existing serves a similar purpose to the surgeon that an instrumental director does in cutting operations; but the accidental forms require cutting, for which operation scissors are preferable to the knife; in some cases both cutting and tearing are are requisite.

VII. There is reason for believing that when there is an accumulation of menstrual fluid within the vagina and uterus, particularly within the latter, that the best time to operate is immediately prior to the menstrual date, as the patency of the newly opened canal is thus better insured.

VIII. Notwithstanding the dangers attending these operations there is good reason for believing that by care and caution, and with a proper use of antiseptics, favorable results may be expected from operations for either congenital or accidental atresias.

